# No association between long-chain *n*-3 fatty acid intake during pregnancy and risk of type 1 diabetes in offspring in two large Scandinavian pregnancy cohorts

**DOI:** 10.1007/s00125-024-06125-4

**Published:** 2024-03-19

**Authors:** Nicolai A. Lund-Blix, Anne A. Bjerregaard, German Tapia, Ketil Størdal, Anne Lise Brantsæter, Marin Strøm, Thorhallur I. Halldorsson, Charlotta Granstrøm, Jannet Svensson, Geir Joner, Torild Skrivarhaug, Pål R. Njølstad, Sjurdur F. Olsen, Lars C. Stene

**Affiliations:** 1 Norwegian Institute of Public Health, Oslo, Norway; 2 Department of Epidemiology Research, Statens Serum Institut, Copenhagen, Denmark; 3 Centre for Clinical Research and Prevention, Copenhagen University Hospitals – Bispebjerg and Frederiksberg, Frederiksberg, Denmark; 4 Faculty of Medicine, Division of Paediatric and Adolescent Medicine, University of Oslo, Oslo, Norway; 5 Division of Paediatric and Adolescent Medicine, Oslo University Hospital, Oslo, Norway; 6 University of the Faroe Islands, Torshavn, Faroe Islands; 7 Faculty of Food Science and Nutrition, University of Iceland, Reykjavik, Iceland; 8 Steno Diabetes Center Copenhagen, Copenhagen, Denmark; 9 Department of Clinical Medicine, University of Copenhagen, Copenhagen, Denmark; 10 Mohn Research Center for Diabetes Precision Medicine, Department of Clinical Science, University of Bergen, Bergen, Norway; 11 Children and Youth Clinic, Haukeland University Hospital, Bergen, Norway; 12 Department of Public Health, University of Copenhagen, Copenhagen, Denmark; 13 Harvard TH Chan School of Public Health, Boston, MA USA

**Keywords:** Child health, Cohort studies, Human, Intrauterine nutrition, Maternal and child health, Nutrition, Pregnancy, Type 1 diabetes

## Abstract

**Aims/hypothesis:**

The aim of this study was to investigate whether higher dietary intake of marine *n*-3 fatty acids during pregnancy is associated with a lower risk of type 1 diabetes in children.

**Methods:**

The Danish National Birth Cohort (DNBC) and the Norwegian Mother, Father and Child Cohort Study (MoBa) together include 153,843 mother–child pairs with prospectively collected data on eicosapentaenoic acid (EPA) and docosahexaenoic acid (DHA) intake during pregnancy from validated food frequency questionnaires. Type 1 diabetes diagnosis in children (*n*=634) was ascertained from national diabetes registries.

**Results:**

There was no association between the sum of EPA and DHA intake during pregnancy and risk of type 1 diabetes in offspring (pooled HR per g/day of intake: 1.00, 95% CI 0.88, 1.14), with consistent results for both the MoBa and the DNBC. Robustness analyses gave very similar results.

**Conclusions/interpretation:**

Initiation of a trial of EPA and DHA during pregnancy to prevent type 1 diabetes in offspring should not be prioritised.

**Graphical Abstract:**

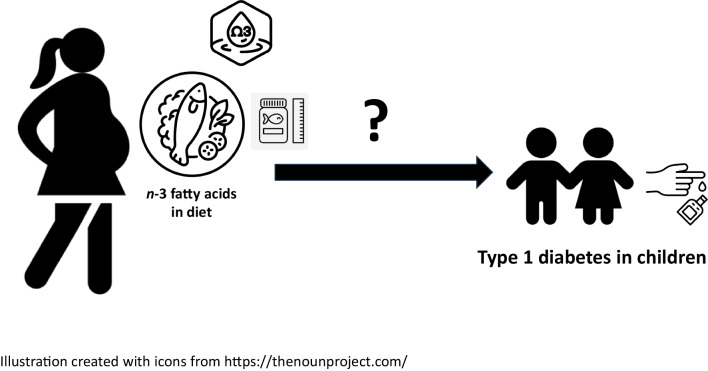

**Supplementary Information:**

The online version contains peer-reviewed but unedited supplementary material available at 10.1007/s00125-024-06125-4.


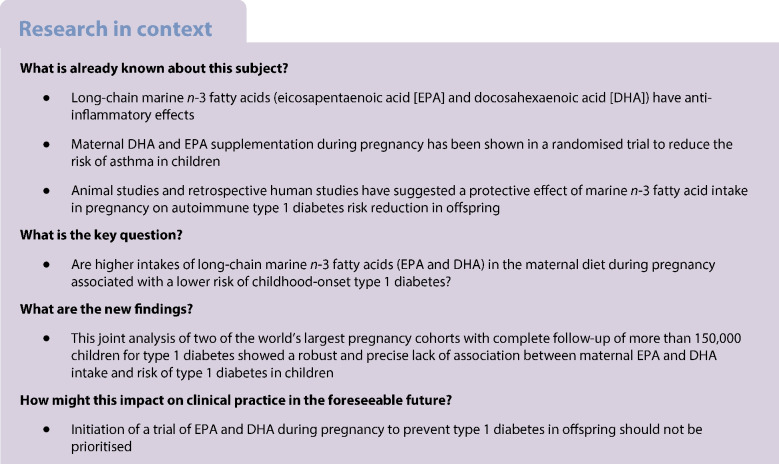


## Introduction

Long-chain marine *n*-3 fatty acids (eicosapentaenoic acid [EPA] and docosahexaenoic acid [DHA]) have anti-inflammatory effects [1] and may influence the development of type 1 diabetes [2]. Studies in experimental animals and retrospective case–control studies have reported associations between early life exposure to *n*-3 fatty acids and lower risk of type 1 diabetes [3]. Furthermore, associations between childhood intake of dietary fatty acids and risk of islet autoimmunity or type 1 diabetes have been reported for high-risk cohorts, although these results were only suggestive for EPA and DHA [2, 4, 5].

DPA, EPA and other fatty acids cross the placenta [6], and maternal dietary intake of these fatty acids during pregnancy may influence offspring physiology and health [7]. Notably, in a randomised trial of a fish oil supplement (containing EPA and DHA) taken from the second trimester of pregnancy, there was a reduction in the risk of asthma or wheezing in children at age 5 years compared with placebo [8], providing proof of concept that maternal DPA and DHA can influence offspring risk of an immune-mediated disease. Few prospective studies have investigated maternal intake of marine *n*-3 fatty acids in relation to risk of childhood-onset type 1 diabetes [9].

We aimed to investigate whether higher intakes of the long-chain marine *n*-3 fatty acids (EPA and DHA) in the maternal diet during pregnancy are associated with a lower risk of childhood-onset type 1 diabetes in two of the largest pregnancy cohorts in the world.

## Methods

### Participants and design

The Danish National Birth Cohort (DNBC) recruited pregnant women from 1996 to 2002 (last birth in 2003) and the Norwegian Mother, Father and Child Cohort Study (MoBa) recruited pregnant women from 1999 to 2008 (last birth in 2009) throughout the respective countries, with 35% and 41% of eligible women participating in the two studies, respectively [10, 11] (see electronic supplementary material [ESM] Methods for further details). Both cohorts are based on written consent and received ethical approval (see ESM Methods).

### Dietary exposure data

**Fig. 1:**
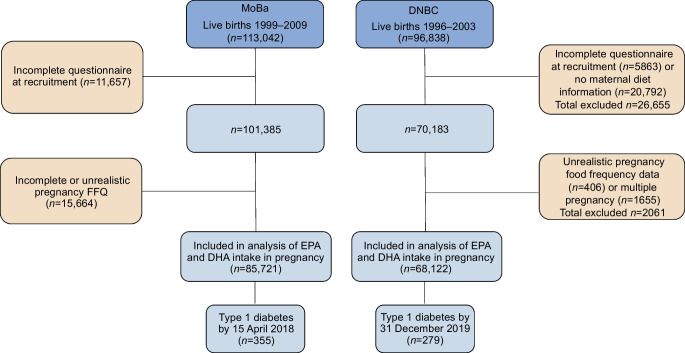
Flow chart of generation of the analysis sample. Total number of mother–child pairs included in the analysis: 153,843 (*n*=634 of whom developed type 1 diabetes during follow-up)

Dietary intake was assessed during pregnancy using validated harmonised food frequency questionnaires (FFQs). In the DNBC, women were recruited during their first antenatal visit to a general practitioner (gestational week ~6), whereas, in the MoBa, pregnant women received an invitation letter prior to their first ultrasound examination in gestational week ~18. Participants from the DNBC included in this study were women who answered an FFQ in mid-pregnancy and participated in two telephone interviews on behavioural factors and health in gestational weeks 12 and 30. The MoBa participants who were included in this study were women who answered a questionnaire on behavioural factors and health in gestational week ~15 and an FFQ in week 22. The MoBa FFQ was introduced from 2002, so data from women recruited before this were excluded from the current analysis (see ESM Methods for further details of the FFQs). The two cohorts together included 153,843 mother–child pairs with data on maternal intake of EPA and DHA (Fig. 1).

### Outcome: type 1 diabetes

We ascertained the date of type 1 diabetes diagnosis for offspring from national childhood diabetes registries (excluding monogenic and type 2 diabetes) (see ESM Methods for further details). Data on offspring were not stratified by sex.

### Statistical analysis

We used time from birth to type 1 diabetes diagnosis in Cox regression analyses, using the sum of EPA and DHA intake from food and supplements during pregnancy as a continuous variable in the primary analyses (estimating HRs per gram per day of intake). Based on the a priori statistical analysis plan the following covariates were included in adjusted models: maternal parity, maternal pre-pregnancy BMI, maternal smoking during pregnancy (never/sometimes/daily), maternal type 1 diabetes, offspring sex, duration of breastfeeding, and maternal education level (see ESM Methods for details of covariates). We pooled the adjusted regression results from the two cohorts using a random-effects meta-analysis (see ESM Methods for further details).

## Results

Among 153,843 children, 634 (0.41%) developed type 1 diabetes during follow-up. The median (IQR) total maternal intake of EPA and DHA together during pregnancy was 0.26 g/day (0.15–0.44) for the DNBC and 0.56 g/day (0.32–1.07) for the MoBa, with a higher intake of fish and a much larger proportion of users of fish oil supplements in Norway (68%) than in Denmark (3.6%; ESM Table 1). Of the women, 46–48% were nulliparous, 9–14% smoked during pregnancy and 68–77% had a pre-pregnancy BMI <25 kg/m^2^ (ESM Table 2).

**Fig. 2:**
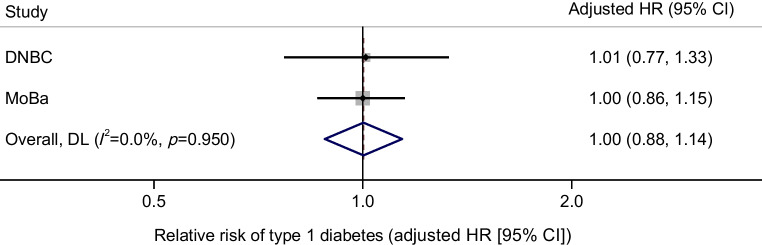
Maternal intake of EPA and DHA during pregnancy and risk of type 1 diabetes in children in the DNBC and MoBa pregnancy cohorts (*n*=153,843 mother–child pairs, of whom *n*=634 children developed type 1 diabetes). HRs per g/day of intake of the sum of EPA and DHA from foods and supplements were adjusted for maternal parity, maternal pre-pregnancy BMI, maternal smoking during pregnancy, maternal type 1 diabetes, offspring sex, duration of breastfeeding, and maternal education level. The *p* value in the plot is for Cochran’s *Q* test for heterogeneity of the HR between the MoBa and the DNBC. DL, DerSimonian and Laird random-effects meta-analysis

There was no association between the sum of EPA and DHA intake during pregnancy and offspring risk of type 1 diabetes (HR 1.00, 95% CI 0.88, 1.14) after adjustment for maternal parity, maternal pre-pregnancy BMI, maternal smoking during pregnancy, maternal type 1 diabetes, offspring sex, duration of breastfeeding, and maternal education level; Fig. 2).

Robustness analyses, including adjustment for additional variables such as maternal vitamin D intake, also supported this conclusion (ESM Table 3). In addition, there was no deviation from linearity or any sign of a threshold effect in categorical analyses; adjusted HRs comparing the upper to the lower quintile of the sum of EPA and DHA intake were 1.19 (95% CI 0.81, 1.74) for the DNBC and 0.84 (0.58, 1.23) for the MoBa (ESM Table 3). Furthermore, secondary analyses showed no association between intake of fish, or the common dietary 18-carbon chain *n*-3 fatty acid alpha-linolenic acid, in pregnancy and risk of type 1 diabetes in offspring (ESM Table 3).

## Discussion

This study found a complete lack of association between EPA and DHA intake during pregnancy and risk of type 1 diabetes; these data were consistent between the DNBC and the MoBa, with relatively narrow 95% CIs.

Our results are consistent with data from a birth cohort with high genetic risk for type 1 diabetes from Finland [9]. Our results therefore confirm and extend this lack of association in larger, general population cohorts. Although not investigating fatty acids directly, another cohort study including children at high genetic risk for type 1 diabetes found no association between maternal fish intake and risk of type 1 diabetes in offspring [12].

The major strengths of our study include the very large, population-based cohorts from two different countries and prospectively collected exposure data during pregnancy, and the complete follow-up of type 1 diabetes diagnosis using nationwide registries. Limitations include the lack of biomarkers of exposure and the low numbers of non-European participants. Below we discuss the strengths and limitations in more detail.

Our cohorts were population based and assessed dietary intake, including dietary supplements, during pregnancy. Biomarker data on EPA and DHA intake were not available in our study; however, the ability of the FFQ to quantify *n*-3 fatty acid intake has been validated against biomarkers in both cohorts [13, 14]. In addition, a nested case–control prospective study from Norway found no association between offspring type 1 diabetes and EPA and DHA levels in the phospholipid fraction of maternal serum collected in late pregnancy [15]. While biomarkers are not influenced by recall and self-report, they are not without problems. Storage and handling of samples may lead to oxidation. Furthermore, biomarkers may be influenced by fasting or recent meals, as well as genetic and other factors regulating metabolism of fatty acids, depending on the type of specimen and assay methods used [16]. Common variants in the fatty acid desaturase (*FADS1*/*FADS2*/*FADS3*) gene cluster are associated with a lower efficiency of conversion of the dietary precursor *n*-3 fatty acid alpha-linolenic acid to EPA and DHA and lower blood levels of EPA and DHA [1]. In the asthma prevention trial cited in the introduction, both lower baseline blood levels of EPA and DHA and a genetic variant associated with lower blood levels of these fatty acids were associated with a stronger relative effect of fish oil supplementation on the prevention of asthma [8]. On the other hand, studies of other disease outcomes in adults have not found consistent interactions between intake of EPA or DHA and genetic variants in the *FADS* gene cluster with regard to cardiometabolic disease outcomes [1]. Future studies of maternal *n*-3 fatty acid intake in pregnancy in relation to childhood type 1 diabetes could consider including genetic variants in the *FADS* gene cluster influencing conversion of alpha-linolenic acid to EPA and DHA.

We cannot exclude the possibility that higher intakes of EPA and DHA than those observed in our studies may show an association with type 1 diabetes. However, analysis of quintiles of the sum of EPA and DHA intake did not suggest any threshold effect. We adjusted for intake of vitamin D in pregnancy in robustness analyses, even though previous evidence suggests no association with type 1 diabetes [17]. Unmeasured confounding, for instance from toxicants in fatty fish, may have influenced our results; however, there is currently no strong evidence supporting an association between such toxicants and type 1 diabetes [2] and we do not believe that toxicants have confounded our results substantially. Given the largely Scandinavian origin of our participants, we believe that our results are generalisable to other European-origin populations, but not necessarily to populations with large proportions of people of other ancestries. Finally, our results do not exclude a potential effect of EPA and DHA intake in children rather than in their pregnant mothers.

In conclusion, the hypothesis that a higher maternal *n*-3 fatty acid intake during pregnancy reduces the risk of type 1 diabetes in offspring was not supported by this study. In a setting where primary prevention trials are extremely expensive and time-consuming [18], we believe that our results, together with the evidence discussed above, clearly indicate that a trial of EPA and DHA during pregnancy to prevent type 1 diabetes in offspring should not be prioritised.

### Supplementary Information

Below is the link to the electronic supplementary material.

Supplementary file1 (PDF 323 KB)

## Data Availability

Computer codes for the statistical analyses and aggregated data are available from the corresponding author on request. The consent given by participants in the two cohorts does not provide for storage of individual-level data in repositories or journals. Access to individual-level data sets from the MoBa database requires an application, approval from the Regional Committee for Medical and Health Research Ethics in Norway, and an agreement with MoBa and the endpoint registries, which can be obtained by applying at https://helsedata.no/en/ (for more information, see the MoBa website, https://www.fhi.no/en/ch/studies/moba/, or email Mor.BarnData@fhi.no). Data from the DNBC used in this study are managed by the DNBC Secretariat at Statens Serum Institut in Copenhagen, Denmark, and can be made available to researchers on approval from the DNBC organisation, compliance with the EU General Data Protection Regulation (GDPR) and approval from the data owner (Statens Serum Institut). Researchers who wish to apply for access to datasets for replication purposes should do so through the DNBC Secretariat (see https://www.dnbc.dk). Access to datasets requires approval from the local data protection agency (in a Danish context; ‘Fortegnelsen’) at the researcher’s institution and an agreement with the DNBC at Statens Serum Institut.

## Abbreviations

DHA: Docosahexaenoic acid
DNBC: Danish National Birth Cohort
EPA: Eicosapentaenoic acid
FFQ: Food frequency questionnaire
MoBa: Norwegian Mother, Father and Child Cohort Study
