# Supplementary material for: No association between long-chain n-3 fatty acid intake during pregnancy and risk of type 1 diabetes in offspring in two large Scandinavian pregnancy cohorts
Source: Diabetologia. 2024 Mar 19;67(6):1023–8. doi: 10.1007/s00125-024-06125-4 (PMC11058590; doi:10.1007/s00125-024-06125-4)
Supplement: Supplementary file 1 — Supplementary file1 (PDF 323 KB) [file 125_2024_6125_MOESM1_ESM.pdf]

Electronic Supplementary Material (ESM)

For the article:

No association between long-chain *n*-3 fatty acid intake during pregnancy and risk of type 1 diabetes in offspring in two large Scandinavian pregnancy cohorts

Nicolai A. Lund-Blix, Anne A. Bjerregaard, German Tapia, Ketil Størdal, Anne Lise Brantsæter, Marin Strøm, Thorhallur I. Halldorsson, Charlotta Granstrøm, Jannet Svensson, Geir Joner, Torild Skrivarhaug, Pål R. Njølstad, Sjurdur F. Olsen and Lars C. Stene

Content

ESM text (methods)........ 2

Population, Study Design and inclusion criteria 2

Dietary exposure data........ 2

Outcome: type 1 diabetes 3

Covariates........ 3

Statistical Analysis 4

ESM Tables........ 5

ESM Table 1. Estimated intake of sum EPA and DHA, fish and alpha-linolenic acid among pregnant women in the DNBC and the MoBa cohorts........ 5

ESM Table 2. Characteristics of study participants in the DNBC and the MoBa cohort. 6

ESM Table 3. Robustness and secondary analyses in the DNBC (*n*=68,122) and the MoBa cohorts (*n*=85,721)[1]........ 7

Questionnaires........ 8

ESM References........ 9

ESM text (methods)

Population, Study Design and inclusion criteria

The Danish National Birth Cohort (DNBC) recruited pregnant women from 1996-2002 and the Norwegian Mother, Father and Child Cohort Study (MoBa) from 1999–2008. These are nation-wide prospective population-based pregnancy cohorts [1, 2]. In Denmark, approximately 35% of all pregnant women were recruited into the DNBC corresponding to 101,042 pregnancies. In MoBa, approximately 41% of all pregnant women were recruited and in total, the cohort includes 95,200 mothers and 114,500 children. Participants in the two studies tended to have higher socioeconomic status than non-participants. While we cannot exclude the possibility of some degree of biased associations due to selection, exposure-disease associations tend to be less affected by selection into studies than prevalence of disease estimates [3-6]. Furthermore, parental socioeconomic status is only weakly or not associated with risk of childhood-onset type 1 diabetes. DNBC and MoBa together include 153,843 mother-child pairs with prospectively collected intake of EPA and DHA during pregnancy included in the current analyses (see Main Figure 1 Flow chart).

Written informed consent was obtained from all participants in both cohorts. The establishment of the cohort and data collection in MoBa were previously based on a license from the Norwegian Data Protection Agency and approval from The Regional Committee for Medical Research Ethics (S-95113/S-97045). They are now based on regulations related to the Norwegian Health Registry Act. The specific research question in the current sub-project was approved by the Regional Ethics Committee South-East C (2013/144/REK). The regional scientific ethics committee for the municipalities of Copenhagen and Frederiksberg approved all DNBC study protocols (27 August 2013: H-2-2013-108). All procedures were in accordance with the Declaration of Helsinki.

In the DNBC, dietary information was available for a total of 70,183 pregnancies. Of those, 406 pregnancies were excluded due to unrealistically low (≤2.5 MJ) or high (>25.0 MJ) energy intake. Exclusion of further 1655 women with multiple pregnancies resulted in 68,122 pregnancies being included in our final analyses. The MoBa held information on 87,070 pregnancies with available maternal dietary information, and 1347 were excluded due to implausible energy intakes. The combined number of mother-child pairs available for analyses from both cohorts was 153,843 (main Figure 1 flow chart)

Dietary exposure data

Dietary assessment methods in both cohorts have been described elsewhere [7]. In both cohorts, the FFQs were designed and validated specifically for pregnant women [8-11], including validation of n-3 fatty acids against biomarkers in both cohorts [8, 10]. Reported frequencies of food intake in the FFQs were scaled into amounts (g/day), by using assumptions of standard portion sizes, gathered from respective national dietary surveys, and by defining recipes for composite foods. The amount of energy and nutrients from each food item was then estimated based on linkage to the respective National Food Composition Table. The total amount of energy, intake of macronutrients and micronutrients was then estimated by aggregating the contribution from all food items. Specifically, long-chain n-3 polyunsaturated fatty acids (LCn3PUFAs) eicosapentaenoic acid (EPA) and docosahexaenoic acid (DHA), alpha-linolenic acid (ALA) and intake of total fish, lean and fatty fish were estimated in g/day. Portion sizes used for fish and fish products including cold cuts and spreads were similar in the two FFQs and the same software for nutrient calculations was used.

In addition, use of fish oil supplements was from the DNBC based on maternal report in structured telephone interviews conducted in gestational week 30 and six months postpartum. We also assessed the intake of all supplements, including fish oils, as reported when the women filled out the FFQ in gestational week 25. In MoBa, use of cod-liver oil/fish oil supplements was reported in the baseline questionnaire answered in gestational week 15 and covered three intervals prior to pregnancy (26-9 weeks, 8-5 weeks, and 4-0 weeks) and four intervals during pregnancy (0-4 weeks, 5-8 weeks, 9-12 weeks, 13+ weeks). In addition, use of supplements was assessed in detail in the FFQ in mid-pregnancy and converted to daily intakes of EPA and DHA based on information about brand, frequency, and amount [12, 13]. Links to questionnaires are provided below.

Outcome: type 1 diabetes

The clinical diagnosis of type 1 diabetes was defined as the first day of insulin treatment in accordance with the EURODIAB criteria. This information was obtained from the Norwegian and the Danish Childhood Diabetes Registries [14, 15] with additional linkage to the Norwegian Patient Registry (NPR) for MoBa participants to confirm the diagnosis. The most updated register linkage was included was end of 2019 for DNBC and April 15, 2018 for MoBa.

Covariates

Based on the a priori statistical analyses plan the following covariates were included in adjusted models: Maternal parity [16, 17], maternal pre-pregnancy body mass index (BMI) [18], maternal smoking during pregnancy (never/sometimes/daily) [19, 20], maternal type 1 diabetes [21, 22], offspring sex [21, 22], duration of breastfeeding [23, 24] and maternal education [25-27] (categorized as in ESM Table 2).

The basis for the selection of covariates was to include in the primary analysis factors relatively robustly associated with risk of type 1 diabetes in previous studies, and at the same time were suspected to be associated with exposure (maternal intake of DHA+EPA) [28]. We also considered availability of reasonably well measured variables and potential bias amplification if including strong predictors of exposure that were not predictors of offspring type 1 diabetes [28, 29]. There were some exceptions from this principle: offspring sex which is associated with offspring type 1 diabetes, but not likely to influence exposure, and breastfeeding duration has not been associated with risk of type 1 diabetes in large prospective studies including MoBa and DNBC [30], but previous experience is that many readers prefer to see analyses adjusted for these variables. Similarly, socioeconomic status indicators such as maternal education is not robustly associated with type 1 diabetes, but is commonly adjusted for [26], and tended to be associated with type 1 diabetes in a large Norwegian cohort [31].

In robustness (sensitivity-) analyses presented in ESM Table 3 we additionally adjusted for the following variables: caesarean section (robustly but weakly associated with type 1 diabetes [22, 32, 33]), birth weight (robustly, but very weakly associated with type 1 diabetes, and likely to be a weak mediator rather than a confounder [34, 35]), maternal energy intake (not known to be associated with type 1 diabetes, but likely positively associated with exposure, and by some considered important to assess nutrient density, although we consider the absolute exposure most relevant in our setting, rather than nutrient density or substitution [36]), maternal intake of vitamin D from the diet and supplements combined (tend to be associated with exposure due to same food sources, and often stated to be associated with risk

of type 1 diabetes despite the clear lack of association between pregnancy- or newborn vitamin D status in MoBa, DNBC [37] and several other large cohort studies [22, 38, 39]).

Statistical Analysis

In our primary analyses the association between maternal intake of LCn3PUFAs and offspring risk of type 1 diabetes was assessed using Cox proportional hazard models to estimate hazard ratios (HRs) with 95% confidence intervals (CIs) in each cohort separately. In absence of previous data to support any particular dose-response relationship, we hypothesized a log-linear association between exposure and outcome and used the exposure as a continuous variable. We adjusted for covariates listed above, and missing information on covariates were imputed using multiple imputation (m=5). Offspring age from birth up to end of 2019 was used in DNBC, and April 15th 2018 for MoBa, as the underlying timescale censoring if death or emigration occurred. Among the MoBa children included in the analysis, the mean and maximum follow-up time was 12.3 years and 16.0 years respectively, and the total number of follow-up time was 1,051,628 person-years. Among the DNBC children included in the analysis, the mean and maximum follow-up time was 17.2 years and 20.4 years respectively, and the total number of follow-up time was 1,171,698 person-years. Because women could participate in the study repeatedly through different pregnancies, we used a robust sandwich covariance matrix estimate to account for interdependent observations. The proportional hazards assumption was evaluated by inspecting the Schoenfeld residuals. We used a random-effects meta-analysis to pool the hazard ratios from the two cohorts [40] using the *metan* package in Stata version 17.

In additional robustness analyses (all presented in ESM Table 3) we assessed the linearity assumption in the primary analysis by also doing categorical analyses with the main exposure divided in fifths (quintiles), and we did complete case analyses only including subjects with no missing data on any covariates.

In secondary analyses (presented in ESM Table 3) the association of maternal intake of fish oil supplements, fatty fish, lean fish, and intake of alpha-linolenic acid were examined in relation to offspring risk of type 1 diabetes.

ESM Tables

ESM Table 1. Estimated intake of sum EPA and DHA, fish and alpha-linolenic acid among pregnant women in the DNBC and the MoBa cohorts

	**DNBC (*n*=68 122)**	**MoBa (*n*=85 721)**
**Sum of EPA and DHA from the diet (g/day)***		
Median (IQR)	0.26 (0.15, 0.44)	0.56 (0.32, 1.07)
Mean (SD)	0.33 (0.29)	0.81 (0.71)
**EPA+DHA from supplements (g/day)†**	(no. of users = 2469)	(no. of users = 58285)
Median (IQR)	2.0 (0.5, 2.8)	0.29 (0.14, 0.80)
Mean (SD)	2.0 (1.7)	0.55 (0.60)
**Fatty fish (g/day)**		
Median (IQR)	0.0 (0.0, 5.3)	8.3 (3.8, 15.6)
Mean (SD)	3.5 (5.7)	12.2 (14.0)
**Lean fish (g/day)**		
Median (IQR)	6.4 (2.5, 11.9)	18.7 (10.8; 27.9)
Mean (SD)	8.3 (8.3)	20.4 (13.5)
**Alpha-linolenic acid (g/d)**		
Median (IQR)	1.9 (2.5)	1.5 (1.1, 1.9)
Mean (SD)	2.1 (0.9)	1.6 (0.7)

* Maternal intakes of EPA and DHA were strongly correlated (Spearman's correlation coefficient=0.98, p <0.001).
† Among users of supplements.
Abbreviations: IQR: Interquartile range (25th percentile, 75th percentile); SD: Standard Deviation; DNBC: Danish National Birth Cohort; MoBa (Norwegian acronym for): Norwegian Mother, Father and Child Cohort Study; EPA: eicosapentaenoic acid; DHA: Docosahexaenoic acid.

ESM Table 2. Characteristics of study participants in the DNBC and the MoBa cohort.

	**DNBC** **(*n*=68 122)**	**MoBa** **(*n*=85 721)**
**Maternal Characteristics**		
Parity [*n*, (%)]		
0	31940 (48%)	39158 (46%)
1	23987 (36%)	30345 (35%)
2 or more	10073 (16%)	16218 (19%)
Missing [*n*]	2122	0
Education [*n*, (%)]		
Less than 9th grade (DNBC)/Less than high school (MoBa)	3426 (7%)	6089 (7%)
10th grade (DNBC)/High school (MoBa)	13212 (26%)	24523 (29%)
Technical school (DNBC)/Up to 4 years of college (MoBa)	7022 (14%)	35043 (41%)
High School or more (DNBC)/More than 4 years of college (MoBa)	27478 (54%)	19677 (23%)
Missing [*n*]	16984	389
Smoking during pregnancy (daily or sometimes) [*n*, (%)]		
No	50987 (76%)	77946 (91%)
Yes	16440 (14%)	7314 (8.6%)
Missing [*n*]	695	461
Maternal type 1 diabetes		
No	67927 (99.7%)	85379 (99.6%)
Yes	195 (0.3%)	342 (0.4%)
Pre-pregnancy BMI [*n*, (%)]		
<20	11002 (18%)	10351 (12%)
20- <25	35314 (59%)	46705 (56%)
25 to <30	12517 (21%)	18420 (22%)
≥ 30	1393 (2%)	8074 (10%)
Missing [*n*]	7896	2171
Breastfeeding duration in months [*n*, (%)]		
<4	10309 (21%)	10330 (14%)
4 - <6	9031 (18%)	6594 (9%)
≥6	29814 (61%)	59218 (78%)
Missing [*n*]	18969	9579
**Offspring characteristics**		
Sex [*n*, (%)]		
Boys	34742 (51%)	43907 (51%)
girls	33380 (49%)	41814 (49%)

ESM Table 3. Robustness and secondary analyses in the DNBC (*n*=68,122) and the MoBa cohorts (*n*=85,721)[1]

	**Unadjusted DNBC**	**Adjusted DNBC[2]**	**Unadjusted MoBa**	**Adjusted MoBa[2]**	**Adjusted, pooled[3]**
	HR (95% CI)	HR (95% CI)	HR (95% CI)	HR (95% CI)	HR (95% CI)
**Primary analyses***					
Sum of EPA and DHA (g/d)	0.98 (0.73, 1.31)	1.01 (0.77, 1.33)	0.98 (0.85, 1.14)	1.00 (0.86, 1.15)	1.00 (0.88, 1.14)
**Categorical analysis to assess potential non-linearity[4]** (Quintiles of sum of EPA and DHA intake)					
1st quintile (reference)	1.00 (reference)	1.00 (reference)	1.00 (reference)	1.00 (reference)	
2nd quintile	1.13 (0.79, 1.61)	1.36 (0.94, 1.95)	0.92 (0.66, 1.27)	0.83 (0.57, 1.20)	
3rd quintile	0.96 (0.66, 1.40)	1.10 (0.74, 1.61)	0.98 (0.71, 1.35)	0.84 (0.58, 1.23)	
4th quintile	0.88 (0.60, 1.29)	1.01 (0.67, 1.50)	1.04 (0.75, 1.42)	0.91 (0.62, 1.33)	
5th quintile	1.02 (0.71, 1.48)	1.19 (0.81, 1.74)	0.89 (0.64, 1.24)	0.84 (0.58, 1.23)	
Robustness analyses:					
Complete case analysis: (DNBC *n*=42221, MoBa *n*= 73707)[2]		0.63 (0.41, 0.98)		0.96 (0.82, 1.13)	0.82 (0.55, 1.22)
Excluding multiple pregnancies in MoBa (n=3119), complete case[2]				0.97 (0.83, 1.14)	
Additional adjustments of primary analysis for					
-Energy intake		0.92 (0.65, 1.30)		0.95 (0.80, 1.12)	0.94 (0.81, 1.10)
-Vitamin D from diet and supplements		0.94 (0.70, 1.27)		0.95 (0.79, 1.14)	0.95 (0.81, 1.11)
-Mode of delivery (vaginal vs Cesarean)		1.01 (0.77, 1.33)		0.95 (0.81, 1.12)	0.97 (0.84, 1.11)
-Birth weight		1.01 (0.76, 1.33)		0.96 (0.81, 1.12)	0.96 (0.84, 1.11)
**Secondary analyses[1]**					
Fish oils Suppl (Yes vs No)	1.32 (0.76, 2.30)	1.34 (0.78, 2.36)	0.84 (0.68, 1.04)	0.79 (0.62, 1.01)	0.97 (0.58, 1.60)
Fatty fish, continuous (per 10 g/d)	0.99 (0.80, 1.22)	1.02 (0.84, 1.25)	1.00 (0.93, 1.08)	0.99 (0.91, 1.08)	1.00 (0.92, 1.08)
Lean fish, continuous (per 10 g/d)	1.05 (0.91, 1.20)	1.07 (0.94, 1.22)	0.98 (0.91, 1.06)	0.96 (0.87, 1.05)	1.01 (0.89, 1.16)
Alpha-linolenic acid (per g/d)	1.03 (0.91, 1.17)	1.04 (0.92, 1.19)	1.07 (0.92, 1.24)	1.04 (0.89, 1.21)	1.04 (0.94, 1.15)

* Same as shown in main Figure 2, for comparison with robustness analyses (sum of EPA and DHA intake during pregnancy, continuous in units g/day), with missing data for covariates imputed as described in ESM Methods.

[1] DNBC (n=68,122, of whom 279 developed type 1 diabetes) and the MoBa cohort (n=85,721 of whom 355 developed type 1 diabetes). [2] Adjusted for maternal pre-pregnancy type 1 diabetes, maternal age, maternal education, maternal body mass index, parity, smoking during pregnancy and duration of breastfeeding without imputation of missing data for covariates. [3] Q-tests for heterogeneity between DNBC and MoBa suggested no significant heterogeneity: Extra adjustment for total energy intake – P(het.)=0.87; Extra adjustment for vitamin D intake – P(Het)=0.95; extra adjustment for mode of delivery - P(het.)=0.71; Extra adjustment for birth weight - P(het.)=0.71; Complete case analysis – P(het.)=0.075; Fish oil supplements (yes/no) - P(het.)=0.087; fatty fish intake - P(het.)=0.76; lean fish intake - P(het.)=0.18; Alpha-linolenic acid – P(het.)=0.99. [4] These results were not pooled because the absolute cut-off values in the two studies differed. The purpose to assess possible deviations from linearity including possible threshold associations is done best by assessing each cohort separately. Quintile cut-offs (and mean) for the sum of EPA and DHA intake (g/d) in MoBa were 1: <0.28 (mean 0.18), 2: 0.28-0.45 (mean 0.36), 3: 0.45-0.72 (mean 0.57), 4: 0.72-1.2 (mean 0.95), 5: ≥1.2 (mean 1.98).

Quintile cut-offs and mean in DNBC were 1: <0.18 (mean 0.12), 2: 0.18-0.30 (mean 0.24), 3: 0.30-0.43 (mean 0.36), 4: 0.43-0.64 (mean 0.52), 5: ≥ 0.64 (mean 0.96)

Abbreviations: DNBC: Danish National Birth Cohort; MoBa (Norwegian acronym for): Norwegian Mother, Father and Child Cohort Study; EPA: eicosapentaenoic acid; DHA: Docosahexaenoic acid; HR: Hazard Ratio; CI: Confidence Interval; het.: heterogeneity

Questionnaires

DNBC
The original questionnaires, detailed descriptions and English translations as well as description of interviews used in DNBC are available here:
https://www.dnbc.dk/data-available

The original food frequency questionnaire (FFQ) used in DNBC is available here (Danish language):
https://www.dnbc.dk/-/media/arkiv/projekt-sites/dnbc/kodeboeger/kostsporegskemaer/ffq_vers7-dansk.pdf

English translation of the DNBC FFQ is available here (18 pages):
https://www.dnbc.dk/-/media/arkiv/projekt-sites/dnbc/kodeboeger/dnbc-food-frequency-questionnaire/dnbc-food-frequency-questionnaire-pdf.pdf

MoBa
All original questionnaires used in MoBa as well as English language translations with explanations and documentation are available here:
https://www.fhi.no/en/ch/studies/moba/for-forskere-artikler/questionnaires-from-moba/

The FFQ used for pregnant women in MoBa is available in original Norwegian language here:
https://www.fhi.no/globalassets/dokumenterfiler/studier/den-norske-mor-far-og-barn--undersokelsenmoba/sporreskjemaer/mor-og-barn-sporreskjema-2-22-svangerskapsuke-versjon-2e.pdf

The MoBa FFQ translated to English here:
https://www.fhi.no/globalassets/dokumenterfiler/studier/den-norske-mor-far-og-barn--undersokelsenmoba/sporreskjemaer/questionnaire---week-22-of-pregnancy.pdf

ESM References

[1] Olsen J, Melbye M, Olsen SF, et al. (2001) The Danish National Birth Cohort – its background, structure and aim. Scand J Public Health 29: 300-307

[2] Magnus P, Birke C, Vejrup K, et al. (2016) Cohort Profile Update: The Norwegian Mother and Child Cohort Study (MoBa). Int J Epidemiol 45: 382-388

[3] Jacobsen TN, Nohr EA, Frydenberg M (2010) Selection by socioeconomic factors into the Danish National Birth Cohort. Eur J Epidemiol 25: 349-355

[4] Nohr EA, Frydenberg M, Henriksen TB, Olsen J (2006) Does low participation in cohort studies induce bias? Epidemiology 17: 413-418

[5] Nilsen RM, Vollset SE, Gjessing HK, et al. (2009) Self-selection and bias in a large prospective pregnancy cohort in Norway. Paediatr Perinat Epidemiol 23: 597-608

[6] Biele G, Gustavson K, Czajkowski NO, et al. (2019) Bias from self selection and loss to follow-up in prospective cohort studies. Eur J Epidemiol 34: 927-938

[7] Olsen SF, Birgisdottir BE, Halldorsson TI, et al. (2014) Possibilities and considerations when merging dietary data from the world's two largest pregnancy cohorts: the Danish National Birth Cohort and the Norwegian Mother and Child Cohort Study. Acta Obstet Gynecol Scand 93: 1131-1140

[8] Mikkelsen TB, Osler M, Olsen SF (2006) Validity of protein, retinol, folic acid and n-3 fatty acid intakes estimated from the food-frequency questionnaire used in the Danish National Birth Cohort. Public Health Nutr 9: 771-778

[9] Brantsæter AL, Haugen M, Alexander J, Meltzer HM (2008) Validity of a new food frequency questionnaire for pregnant women in the Norwegian Mother and Child Cohort Study (MoBa). Maternal & child nutrition 4: 28-43

[10] Brantsæter AL, Haugen M, Hagve TA, et al. (2007) Self-reported dietary supplement use is confirmed by biological markers in the Norwegian Mother and Child Cohort Study (MoBa). Ann Nutr Metab 51: 146-154

[11] Meltzer HM, Brantsaeter AL, Ydersbond TA, Alexander J, Haugen M (2008) Methodological challenges when monitoring the diet of pregnant women in a large study: experiences from the Norwegian Mother and Child Cohort Study (MoBa). Maternal & child nutrition 4: 14-27

[12] Brantsæter AL, Englund-Ögge L, Haugen M, et al. (2017) Maternal intake of seafood and supplementary long chain n-3 poly-unsaturated fatty acids and preterm delivery. BMC pregnancy and childbirth 17: 41

[13] Haugen M, Brantsæter AL, Alexander J, Meltzer HM (2008) Dietary supplements contribute substantially to the total nutrient intake in pregnant Norwegian women. Ann Nutr Metab 52: 272-280

[14] Skrivarhaug T, Stene LC, Drivvoll AK, Strøm H, Joner G (2014) Incidence of type 1 diabetes in Norway among children aged 0-14 years between 1989 and 2012: has the incidence stopped rising? Results from the Norwegian Childhood Diabetes Registry. Diabetologia 57: 57-62

[15] Svensson J, Cerqueira C, Kjaersgaard P, et al. (2016) Danish Registry of Childhood and Adolescent Diabetes. Clin Epidemiol 8: 679-683

[16] Stene LC, Magnus P, Lie RT, Søvik O, Joner G, the Norwegian Childhood Diabetes Study Group (2001) Maternal and paternal age at delivery, birth order, and risk of childhood onset type 1 diabetes: population based cohort study. BMJ 323: 369-371

[17] Cardwell CR, Stene LC, Joner G, et al. (2011) Birth order and childhood type 1 diabetes risk: a pooled analysis of 31 observational studies. Int J Epidemiol 40: 363-374

[18] Magnus MC, Olsen SF, Granström C, et al. (2018) Paternal and maternal obesity but not gestational weight gain is associated with type 1 diabetes. Int J Epidemiol 47: 417-426

[19] Magnus MC, Tapia G, Olsen SF, et al. (2018) Parental smoking and risk of childhood-onset type 1 diabetes. Epidemiology 29: 848-856

[20] Wei Y, Andersson T, Edstorp J, et al. (2022) Maternal smoking during pregnancy and type 1 diabetes in the offspring: a nationwide register-based study with family-based designs. BMC Med 20: 240
[21] Atkinson MA, Eisenbarth GS, Michels AW (2014) Type 1 diabetes. Lancet 383: 69-82
[22] Norris JM, Johnson RK, Stene LC (2020) Type 1 diabetes-early life origins and changing epidemiology. Lancet Diabetes Endocrinol 8: 226-238
[23] Hakola L, Takkinen HM, Niinistö S, et al. (2018) Infant feeding in relation to the risk of advanced islet autoimmunity and type 1 diabetes in children with increased genetic susceptibility: a cohort study. Am J Epidemiol 187: 34-44
[24] Cardwell CR, Stene LC, Ludvigsson J, et al. (2012) Breast-feeding and childhood-onset type 1 diabetes: a pooled analysis of individual participant data from 43 observational studies. Diabetes Care 35: 2215-2225
[25] Lopez-Doriga Ruiz P, Tapia G, Bakken IJ, et al. (2024) Parental education and occupation in relation to childhood type 1 diabetes: nationwide cohort study. J Epidemiol Community Health
[26] Lopez-Doriga Ruiz P, Stene LC (2023) Is socio-economic status associated with risk of childhood type 1 diabetes? Literature review. Diabet Med 40: e15182
[27] Prætorius K, Urhoj SK, Andersen AN (2023) Parental socio-economic position and the risk of type 1 diabetes in children and young adults in Denmark: A nation-wide register-based study. Scand J Public Health 51: 944-952
[28] Lash TL, VanderWeele TJ, Haneuse S, Rothman KJ (2021) Modern epidemiology. Wolters Kluwer, Philadelpia
[29] Pearl J (2011) Invited commentary: understanding bias amplification. Am J Epidemiol 174: 1223-1227; discussion pg 1228-1229
[30] Lund-Blix NA, Dydensborg Sander S, Størdal K, et al. (2017) Infant feeding and risk of type 1 diabetes in two large Scandinavian birth cohorts. Diabetes Care 40: 920-927
[31] Lopez-Doriga Ruiz P, Tapia G, Bakken IJ, et al. (2022) Parental education and occupation in relation to risk of childhood type 1 diabetes: nationwide cohort study. medRxiv: 2022.2007.2026.22277835
[32] Cardwell CR, Stene LC, Joner G, et al. (2008) Caesarean section is associated with an increased risk of childhood onset type 1 diabetes: A meta-analysis of observational studies. Diabetologia 51: 726-735
[33] Metsälä J, Hakola L, Lundqvist A, Virta LJ, Gissler M, Virtanen SM (2020) Perinatal factors and the risk of type 1 diabetes in childhood and adolescence-A register-based case-cohort study in Finland, years 1987 to 2009. Pediatric diabetes 21: 586-596
[34] Stene LC, Magnus P, Lie RT, Søvik O, Joner G, the Norwegian Childhood Diabetes Study Group (2001) Birth weight and childhood onset type 1 diabetes: population based cohort study. BMJ 322: 889-892
[35] Cardwell CR, Stene LC, Joner G, et al. (2010) Birthweight and the risk of childhood-onset type 1 diabetes: a meta-analysis of observational studies using individual patient data. Diabetologia 53: 641-651
[36] Tomova GD, Arnold KF, Gilthorpe MS, Tennant PWG (2022) Adjustment for energy intake in nutritional research: a causal inference perspective. The American journal of clinical nutrition 115: 189-198
[37] Thorsen SU, Mårild K, Olsen SF, et al. (2018) Lack of association between maternal or neonatal vitamin D status and risk of childhood type 1 diabetes: a Scandinavian case-cohort study. Am J Epidemiol 187: 1174-1181
[38] Jacobsen R, Thorsen SU, Cohen AS, et al. (2016) Neonatal vitamin D status is not associated with later risk of type 1 diabetes: results from two large Danish population-based studies. Diabetologia 59: 1871-1881
[39] Mäkinen M, Löyttyniemi E, Koskinen M, et al. (2019) Serum 25-hydroxyvitamin D concentrations at birth in children screened for HLA-DQB1 conferred risk for type 1 diabetes. J Clin Endocrinol Metab 104: 2277-2285

[40] DerSimonian R, Laird N (1986) Meta-analysis in clinical trials. Control Clin Trials 7: 177-188
